# Devise Plans for Regulating Practice of Medicine

**Published:** 1908-07

**Authors:** 


### TO DEVISE PLANS FOR REGULATING PRACTICE OF MEDICINE.

A very important meeting was held in Macon, June 10th, 1908. Those invited were the Deans of Medical Colleges of the State, the Board of Medical Examiners, the President of the Georgia Medical Association and the Chairman of the Councillors of the State Association. The object of this meeting is to consider and discuss ways and means to regulate the practice of medicine in Georgia. This movement will undoubtedly meet the approval of every legal practitioner in the State, and the Journal-Record will heartily co-operate in any possible plan that may be decided upon.

